# Brain Reward System And Its Volumetric Investigations In Alcohol Addiction

**DOI:** 10.1192/j.eurpsy.2023.1935

**Published:** 2023-07-19

**Authors:** M. F. Tabara, M. Atmaca, H. Yildirim, O. Solmaz, M. A. Essibayi

**Affiliations:** 1 Psychiatry, Bingol State Hospital, bingol; 2 Psychiatry; 3 Radiology; 4 Firat University, elazig, Türkiye

## Abstract

**Introduction:**

Alcohol use disorder (AUD) is a diagnosis that includes both addiction and abuse concepts that entered our lives with the DSM-5. The prevalence of AUD is 8.1% in men and 1.7% in women in Türkiye, and it is getting more and more common. Biopsychosocial factors play a role in the etiology of AUD.

**Objectives:**

The brain reward system, which includes many cortical and subcortical structures, plays an active role in the initiation and maintenance of alcohol dependence. In this study, we aimed to reveal the structural changes in alcohol dependence.

**Methods:**

15 cases with AUD and 17 healthy controls were compared in terms of total white matter, total gray matter, nucleus accumbens, amygdala and hippocampus volumes. AUDIT, MAST and alcohol addiction severity scale were administered to all participants. Magnetic resonance imaging of all participants was performed. Then, the relevant regions were painted cross-sectionally and volume measurements were made. The case group was evaluated for the diagnosis of AUD with SCID-V. Volume averages were evaluated with Student’s t test. ANCOVA was used to remove confounding factors and re-evaluate the difference between volumes.

**Results:**

Although there were differences between volumes in the first analyzes with Student’s t test, they were not statistically significant.

Age and gender variables, which were determined by the literature to have an effect on volume measurements, were re-evaluated with the ANCOVA test. When the effects of age and gender variables were removed in the evaluation, the right hippocampus volume was found to be significantly reduced in the AUD group compared to the control group (F=5.26, p=0.03). Again, no significant difference was observed in the two groups in terms of areas other than the volume of the right hippocampus. Pearson correlation analysis was used to evaluate the relationship between scale scores, duration of alcohol use and amount taken, and volumetric measurements, but no statistically significant relationship was found.

**Conclusions:**

Different findings have been reported in the literature regarding the examined region volumes. Our study found volumetric changes consistent with most previous studies. For more generalizable results, studies with a large number of participants are needed.

**Disclosure of Interest:**

None Declared

